# Trends of rehabilitation needs in 195 countries and regions from 1990 to 2021 and the impact of the COVID-19 pandemic on it: a systematic analysis of the Global Burden of Disease Study 2021

**DOI:** 10.1097/JS9.0000000000003486

**Published:** 2025-09-09

**Authors:** Houqiang Zhang, Zhengwei Chen, Shufen Liu, Weihong Shi, Xingyu Cui, Wangshu Yuan, Runlin Shi, Yuanchun Zhu, Xiaoyi Zhao, Xiaotian Chu, Lixia Chen, Qing Li

**Affiliations:** a Department of Rehabilitation Medicine, Peking Union Medical College Hospital, Chinese Academy of Medical Sciences and Peking Union Medical College, Beijing, China; b Institute of Nursing and Rehabilitation, North China University of Science and Technology, Tangshan, Hebei Province, China

**Keywords:** global burden of disease, prevalence, rehabilitation needs, sociodemographic index, universal health coverage effective index, years lived with disability

## Abstract

**Background:**

The magnitude and composition of global rehabilitation needs are evolving, particularly following the COVID-19 pandemic. Our study aims to systematically analyze the trends of rehabilitation needs across 195 countries and regions from 1990 to 2021 and to evaluate the impact of the COVID-19 pandemic on global rehabilitation needs.

**Materials and methods:**

This study included 27 health conditions for which rehabilitation is considered a key intervention, with all data from the Global Burden of Disease Study 2021. A multidimensional analytical framework was employed to analyze multiple aspects of rehabilitation needs, including segmented regression, smoothing spline models, decomposition analysis, inequality assessment, frontier analysis, and the Bayesian age–period–cohort model.

**Results:**

Due to the impact of the COVID-19 pandemic, the global number of people in need of rehabilitation reached 2.56 billion in 2021, up significantly from 2.41 billion in 2019. Over the past three decades, rehabilitation needs have increased notably for neurological disorders, sensory impairments, chronic respiratory diseases, cardiovascular diseases, and neoplasms. Notably, when a country’s universal health coverage effective index reaches approximately 80, the rehabilitation needs tend to stabilize or even decline. The increasing needs for rehabilitation services are primarily driven by population growth and population aging. In 2021, rehabilitation needs remained concentrated in high Sociodemographic Index (SDI) regions, but it showed a trend toward greater global equity. Frontier analysis indicated that a reduction in the burden of rehabilitation-related conditions is associated with improvements in social development. Nonetheless, certain high-SDI countries exhibited significant deviations from this trend. By 2035, the absolute number of prevalent cases with rehabilitation-related conditions is projected to reach approximately 2.64 billion.

**Conclusion:**

The need for rehabilitation services has increased significantly worldwide, especially following the COVID-19 pandemic. This trend places greater demands on the allocation, equity, and accessibility of global health resources.

**HIGHLIGHTS**

- A sharp increase in rehabilitation needs due to the COVID-19 pandemic: In 2021, the global number of people in need of rehabilitation reached 2.56 billion, up significantly from 2.41 billion in 2019.
- Over the past three decades, rehabilitation needs have increased notably for neurological disorders, sensory impairments, chronic respiratory diseases, cardiovascular diseases, and neoplasms.
- This study provides new insights into the relationship between rehabilitation and universal health coverage (UHC): rehabilitation services are a vital component of achieving universal health, while improvements in UHC levels contribute to reducing the overall burden of rehabilitation-related conditions.
- The rising demand for rehabilitation is primarily driven by population growth and population aging, though trends vary substantially across regions. Therefore, addressing the global disparities in rehabilitation burden requires the development of differentiated strategies that consider each country’s population structure, disease profile, level of social development, and national context.
- Digital rehabilitation and tele-rehabilitation may help expand access to services and support the global development of rehabilitation systems.

## Introduction

Rehabilitation encompasses a range of evidence-based interventions intended to improve functional abilities and reduce physical disability while considering the dynamic interaction between health conditions and environmental factors. It plays a critical role in enhancing patients’ well-being while reducing the broader socioeconomic impact on families and healthcare systems^[1]^. Due to the global population aging, the proportion of individuals aged 60 and over is expected to nearly double – from 12% to 22% by 2050^[2]^. This demographic transition is projected to drive the sustained rise in chronic conditions such as arthritis^[3]^, stroke^[4]^, and cancer^[5]^, along with an increasing incidence of injury^[6]^, resulting in the growing global demand for rehabilitation services.

Rehabilitation is recognized as an integral element of universal health coverage (UHC) and a critical strategy for achieving Sustainable Development Goal 3, which aims to ensure individuals’ health and well-being across all ages^[7]^. However, the current global distribution of rehabilitation resources is extremely uneven. Although high-income countries generally have mature rehabilitation systems and adequate professional staffing, many of low- and middle-income countries are facing the challenge of resource shortages^[8,9]^. This disparity has limited access to timely and appropriate rehabilitation care for individuals. Advancing global rehabilitation efforts requires a clear understanding of regional disparities of rehabilitation needs and effective strategies to address the imbalance between growing demand and insufficient services, thereby enabling the rational allocation and efficient utilization of rehabilitation resources.

The World Health Organization (WHO) and the Institute for Health Metrics and Evaluation (IHME) jointly conducted the first global assessment of rehabilitation needs, estimating that approximately 2.41 billion patients worldwide benefited from rehabilitation in 2019^[10]^. The study offered critical evidence to inform national rehabilitation strategies. However, with the constant shift of global demographics and disease patterns, the magnitude and composition of rehabilitation needs are also evolving.

Drawing on the latest data from the Global Burden of Disease Study (GBD) 2021, this study employed a multidimensional analytical framework to comprehensively assess rehabilitation needs in 195 countries and regions. To the best of our knowledge, this is the first study to capture the potential changes in rehabilitation needs following the COVID-19 pandemic and explore the nonlinear relationship between rehabilitation needs and the UHC effective index. In this study, we are expected to offer a more practical and forward-looking foundation for the scientific allocation of rehabilitation resources, policy development, and the optimization of service delivery systems worldwide. This cross-sectional study has been reported in line with the STROCSS guidelines^[11]^.

## Methods

### Data sources

The data used in this cross-sectional study were obtained from the WHO Rehabilitation Need Estimator (https://vizhub.healthdata.org/rehabilitation/), which was jointly developed by WHO and IHME based on the GBD 2021^[12]^. It covered data from 195 countries and organized them across seven regions, including high-income countries defined by the World Bank and the six regions classified by WHO. The tool presents both absolute numbers and age-standardized rates of prevalence and years lived with disability (YLDs) for 27 health conditions, divided into eight major categories (Supplemental Digital Content Table 1, available at: http://links.lww.com/JS9/F72). The selection process for rehabilitation-related conditions was as follows: First, the top 20 conditions with the highest YLDs were identified. Second, the conditions would have been excluded if rehabilitation were only an adjunct intervention to treat them (e.g. dietary iron deficiency, oral disorders). Finally, WHO convened a panel of rehabilitation experts to review the preliminary list and add conditions for which rehabilitation is considered a key treatment. In comparison with GBD 2019, this study has included long COVID and neural tube defects as newly identified rehabilitation-related conditions, improving the accuracy and completeness of global estimates.

The objective of UHC is to ensure that all individuals who have rehabilitation needs receive health services without suffering financial hardship. Based on the GBD 2019 Study, we obtained the UHC effective coverage index (Supplemental Digital Content Table 2, available at: http://links.lww.com/JS9/F72), which serves as a composite indicator to assess the quality of preventive, promotive, and therapeutic health services^[13]^. This index ranges from 0 to 100, with higher scores indicating better performance in providing health services for the public.

In addition, to quantify the level of sociodemographic development across countries, we have employed the Sociodemographic Index (SDI) as a measurement tool (Supplemental Digital Content Table 3, available at: http://links.lww.com/JS9/F72). The calculation of SDI included the geometric mean of three standardized components: total fertility rate under the age of 25, the mean of the years of individuals receiving education among individuals aged 15 and older, and lag-distributed income per capita. The SDI ranges from 0 to 1, with higher values indicating a higher level of socioeconomic development in the given country or region^[14]^.

### Statistical methods

The indicators used to assess rehabilitation needs included the number of prevalence and YLDs, as well as their age-standardized rates, each accompanied by the 95% uncertainty interval (UI). A log-transformed linear regression model was employed to estimate the Estimated Annual Percentage Change (EAPC) and its 95% confidence interval (CI) in age-standardized prevalence and YLD rates for rehabilitation-related conditions from 1990 to 2021, thereby quantifying overall temporal trends. Subsequently, segmented regression was applied to identify annual percent change (APC) within distinct time periods, which aims to detect trend inflection points and provide a detailed interpretation of acceleration or deceleration across different stages.

To assess the nonlinear association between rehabilitation needs and the UHC effective index, we have applied smoothing spline models to fit the smooth curve, which could automatically determine the number and location of knots and the polynomial degree based on the data distribution and span parameter. Additionally, Spearman correlation analysis was performed to calculate the correlation coefficient (*r*) and corresponding *P*-value between age-standardized rates and the UHC effective index to examine the significance of their relationship.

In the assessment of drivers, we have employed the Das Gupta decomposition method to divide changes in rehabilitation needs into three components: population growth, aging, and epidemiological changes, which aim to quantify the relative contribution of each factor. We also used the Slope Index of Inequality (SII) and the Concentration Index, as defined by the WHO, to measure the absolute and relative inequalities in the distribution of rehabilitation needs across countries or regions^[15]^. Additionally, through frontier analysis^[16]^, we have evaluated the theoretically optimal level of rehabilitation needs for countries at comparable development levels and examined the gap between the optimal need and the actual need, identifying potential areas for the improvement of resource allocation and service efficiency.

Finally, the study projected future global trends in rehabilitation needs using the Bayesian Age-Period-Cohort (BAPC) model, which employed the Integrated Nested Laplace Approximation method to approximate the marginal posterior distributions^[17]^. This approach effectively avoids the mixing and convergence issues associated with Markov Chain Monte Carlo methods while maintaining computational efficiency. All analyses were conducted in the R 4.3.3 environment, with a significance level set at *P* < 0.05.

## Results

The global number of individuals with conditions requiring rehabilitation increased markedly, rising from 1.53 (95% UI: 1.49–1.59) billion in 1990 to 2.56 (95% UI: 2.49–2.65) billion in 2021. Correspondingly, YLDs associated with rehabilitation steadily rose from 190.73 (95% UI: 145.85–244.34) million in 1990 to 342.09 (95% UI: 263.91–438.10) million in 2021. Despite the continuous rise in the absolute need for rehabilitation services, both the global age-standardized prevalence and YLD rates of rehabilitation-related conditions showed a downward trend during this period, with EAPCs of −0.23 (95% CI: −0.25 to −0.21) and −0.16 (95% CI: −0.19 to −0.13), respectively (Table 1).

**Table 1:** Rehabilitation needs in 2021 and trends between 1990 and 2021 by World Health Organization regions.

Regions	Prevalence			YLDs		
	Number (95% UI)	ASRs per 100 000 persons (95% UI)	EAPC (95% CI)	Number (95% UI)	ASRs per 100 000 persons (95% UI)	EAPC (95% CI)
Global	2 562 527 297 (2 490 617 974, 2 653 665 631)	30752.20 (29865.77, 31874.30)	−0.23 (−0.25, −0.21)	342 090 054 (263 908 584, 438 098 160)	4113.21 (3176.89, 5257.20)	−0.16 (−0.19, −0.13)
World Bank High Income	536 175 174 (523 283 379, 550 299 297)	33253.17 (32363.57, 34292.11)	−0.28 (−0.29, −0.26)	74 028 219 (57 287 792, 96 221 190)	4233.10 (3281.05, 5459.95)	−0.22 (−0.23, −0.21)
Western Pacific Region	560 285 987 (543 404 363, 578 221 436)	26545.56 (25778.85, 27347.02)	−0.07 (−0.11, −0.02)	75 514 721 (57 422 444, 97 258 864)	3581.63 (2741.42, 4603.40)	−0.05 (−0.09, −0.01)
South-East Asia Region	628 532 239 (605 734 916, 655 903 308)	31086.35 (29994.18, 32375.52)	−0.21 (−0.23, −0.19)	84 154 007 (64 799 460, 107 471 130)	4279.20 (3308.60, 5444.79)	−0.20 (−0.24, −0.16)
European Region	168 019 536 (163 363 432, 173 143 480)	35409.48 (34338.59, 36608.05)	−0.40 (−0.45, −0.36)	22 258 773 (17 050 998, 28 561 055)	4588.78 (3521.56, 5873.59)	−0.37 (−0.43, −0.31)
Eastern Mediterranean Region	192 730 899 (185 491 617, 202 029 466)	32583.17 (31512.23, 33894.38)	−0.10 (−0.12, −0.09)	25 278 566 (19 359 082, 32 453 301)	4491.89 (3455.51, 5716.75)	−0.10 (−0.13, −0.06)
Region of the Americas	215 340 555 (209 381 678, 222 552 669)	32601.71 (31720.25, 33698.99)	−0.20 (−0.24, −0.16)	26 639 288 (20 432 061, 34 203 697)	4054.90 (3114.35, 5199.82)	−0.15 (−0.19, −0.11)
African Region	238 919 820 (229 708 086, 251 027 189)	28528.63 (27613.96, 29671.92)	−0.12 (−0.15, −0.09)	32 578 987 (24 912 102, 41 992 389)	4075.39 (3125.69, 5211.01)	−0.04 (−0.10, 0.01)

At the regional level, the total number of cases in the South-East Asia Region and the Western Pacific Region had exceeded those in high-income countries by 2021 (Table 1). After adjusting for age structure, trends in prevalence and YLDs across regions were generally consistent with the global pattern, showing a widespread decline. Among these, the European Region showed the most pronounced decrease, with EAPCs for the age-standardized prevalence and YLD rates of −0.40 (95% CI: −0.45 to −0.36) and −0.37 (95% CI: −0.43 to −0.31), respectively, though levels remained the highest globally. In contrast, the Western Pacific Region showed only minor declines, with EAPCs of −0.07 (95% CI: −0.11 to −0.02) and −0.05 (95% CI: −0.09 to −0.01), reflecting persistent and complex rehabilitation needs (Table 1).

Further analysis of trends and regional distribution for the eight major categories of rehabilitation-related conditions revealed increasing burdens for most conditions, including neurological disorders, sensory impairments, chronic respiratory diseases, cardiovascular diseases, and neoplasms. In contrast, the burden of musculoskeletal disorders and mental disorders showed a declining trend (Table 2). Notably, in 2021, the South-East Asia Region had the highest global burden for four categories: neurological disorders, sensory impairments, mental disorders, and chronic respiratory diseases, highlighting serious challenges in managing various rehabilitation-related conditions in this region (Table 2).

**Table 2:** Burden of eight major rehabilitation-related diseases and highest-burden regions in 2021.

All condition categories	Prevalence			YLDs			Region with the highest age-standardized prevalence/YLD rates in 2021
	Number (95% UI)	ASRs per 100 000 persons (95%UI)	EAPC (95% CI)	Number (95% UI)	ASRs per 100 000 persons (95%UI)	EAPC (95% CI)	
Musculoskeletal disorders	1 693 808 277 (1 608 023 292, 1 777 807 497)	20084.23 (19063.63, 21083.12)	−0.45 (−0.46, −0.44)	153 458 168 (111 409 520, 210 890 543)	1816.39 (1320.59, 2486.14)	−0.46 (−0.48, −0.44)	European Region
Neurological disorders	225 382 920 (215 836 356, 235 207 819)	2758.37 (2644.02, 2878.23)	0.17 (0.15, 0.19)	52 346 199 (37 572 115, 67 457 539)	640.50 (459.39, 824.44)	0.13 (0.11, 0.15)	South-East Asia Region
Sensory impairments	737 411 885 (683 609 549, 788 994 754)	8777.10 (8154.07, 9364.09)	0.05 (0.04, 0.07)	56 146 712 (38 945 114, 76 676 133)	670.96 (465.24, 913.53)	−0.10 (−0.12, −0.08)	South-East Asia Region
Mental disorders	224 024 112 (183 516 537, 265 147 311)	2869.83 (2340.49, 3405.24)	−0.19 (−0.22, −0.16)	36 575 488 (29 097 778, 44 147 924)	457.78 (365.21, 554.03)	−0.02 (−0.03, −0.01)	South-East Asia Region
Chronic respiratory diseases	123 519 489 (111 464 914, 137 245 464)	1452.28 (1310.85, 1607.35)	0.03 (−0.01, 0.07)	14 857 505 (12 405 349, 17 130 013)	174.36 (145.41, 201.05)	0.13 (0.07, 0.19)	South-East Asia Region
Cardiovascular diseases	36 756 667 (32 398 428, 42 049 586)	446.97 (394.56, 508.61)	0.17 (0.16, 0.18)	4 193 417 (2 823 101, 5 879 077)	51.02 (34.33, 71.39)	0.18 (0.17, 0.19)	Eastern Mediterranean Region
Neoplasms	143 978 077 (132 650 845, 156 559 024)	1697.91 (1562.48, 1846.76)	0.27 (0.22, 0.31)	6 321 373 (4 682 693, 8 264 424)	73.45 (54.47, 96.07)	0.21 (0.14, 0.27)	World Bank High Income
COVID-19	60 655 776 (26 970 651, 120 668 936)	749.39 (330.41, 1497.08)	NA	13 329 483 (4 148 982, 32 666 391)	164.87 (50.64, 405.98)	NA	African Region

**Figure 1.**
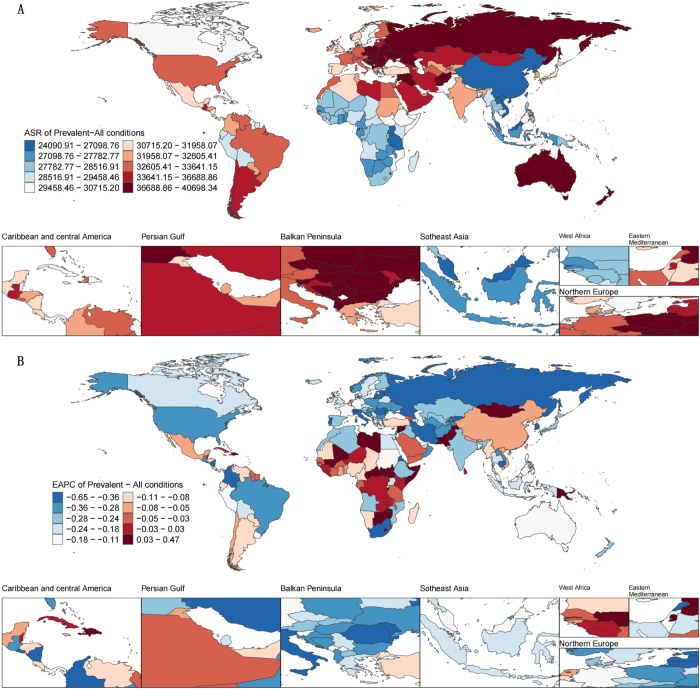
Prevalence estimates for rehabilitation-related conditions worldwide. (A) Age-standardized prevalence rates of rehabilitation-related conditions in countries worldwide in 2021. (B) EAPC of prevalence from 1990 to 2021.

**Figure 2.**
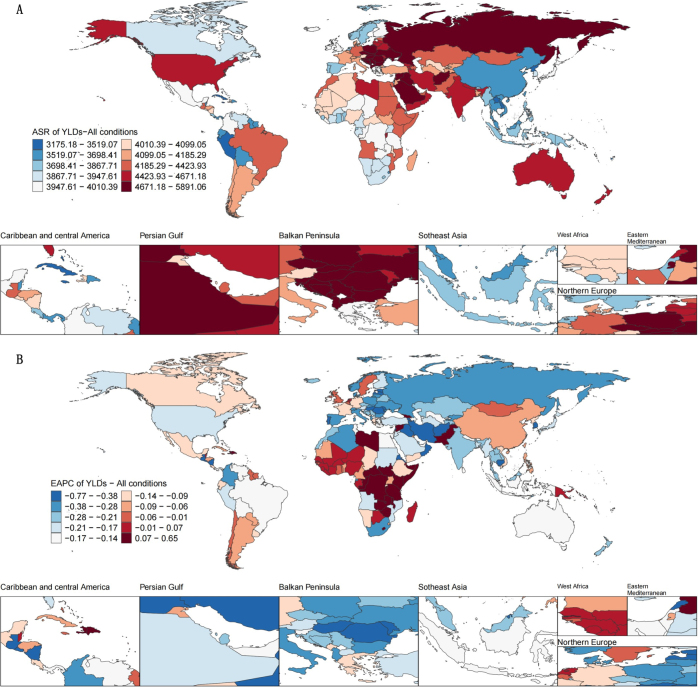
YLDs estimates for rehabilitation-related conditions worldwide. (A) age-standardized YLD rates of rehabilitation-related conditions in countries worldwide in 2021. (B) EAPC of YLDs from 1990 to 2021.

At the national level, in 2021, the highest age-standardized prevalence rates of rehabilitation-related conditions were observed in Afghanistan (40295.38 per 100 000 population; 95% UI: 37464.69–44587.79), Albania (39456.85; 95% UI: 38171.24–40996.48), and Bulgaria (39385.74; 95% UI: 38129.65–40780.08) (Fig. 1). From 1990 to 2021, 30 countries exhibited increasing trends in age-standardized prevalence rates, with the fastest growth in Burundi (EAPC = 0.47; 95% CI: 0.27–0.68), followed by the Syrian Arab Republic (EAPC = 0.45; 95% CI: 0.30–0.61) and Haiti (EAPC = 0.43; 95% CI: 0.28–0.58) (Fig. 1). In 2021, the highest age-standardized YLD rates were also observed in Afghanistan (5832.73 per 100 000; 95% UI: 4351.12–7733.97), Czechia (5105.00; 95% UI: 3917.88–6557.02), and Bulgaria (5063.02; 95% UI: 3838.06–6604.59) (Fig. 2). A total of 38 countries showed increasing age-standardized YLD rates during this period, with Burundi again leading (EAPC = 0.65; 95% CI: 0.43–0.88), followed by Haiti (EAPC = 0.47; 95% CI: 0.27–0.67) and the Syrian Arab Republic (EAPC = 0.28; 95% CI: 0.13–0.44) (Fig. 2).

**Figure 3.**
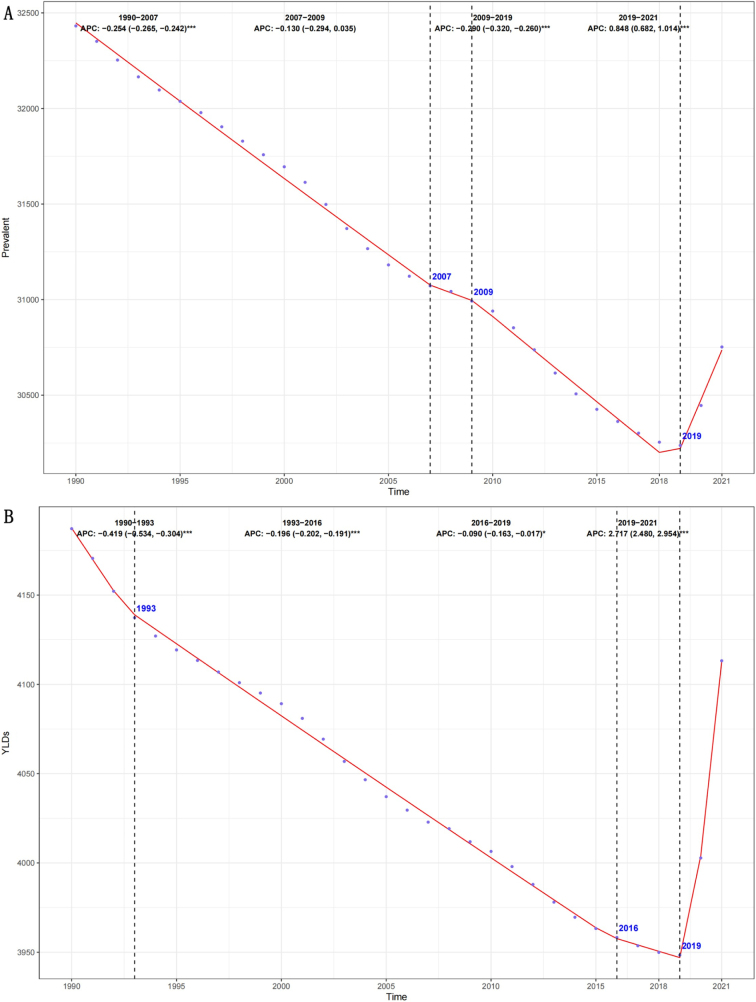
Annual percent change (APC) and trends in global prevalence and YLDs of rehabilitation-related conditions from 1990 to 2021. (A) Prevalence rate. (B) YLD rate.

Using segmented regression, we analyzed global trends in age-standardized prevalence and YLD rates from 1990 to 2021 (Fig. 3). While both indicators showed an overall declining trend, a notable reversal occurred after 2019. Specifically, between 2019 and 2021, the APC in prevalence was 0.848 (95% CI: 0.682–1.014), while the APC for YLDs was even more substantial at 2.717 (95% CI: 2.480–2.954) (Fig. 3).

**Figure 4.**
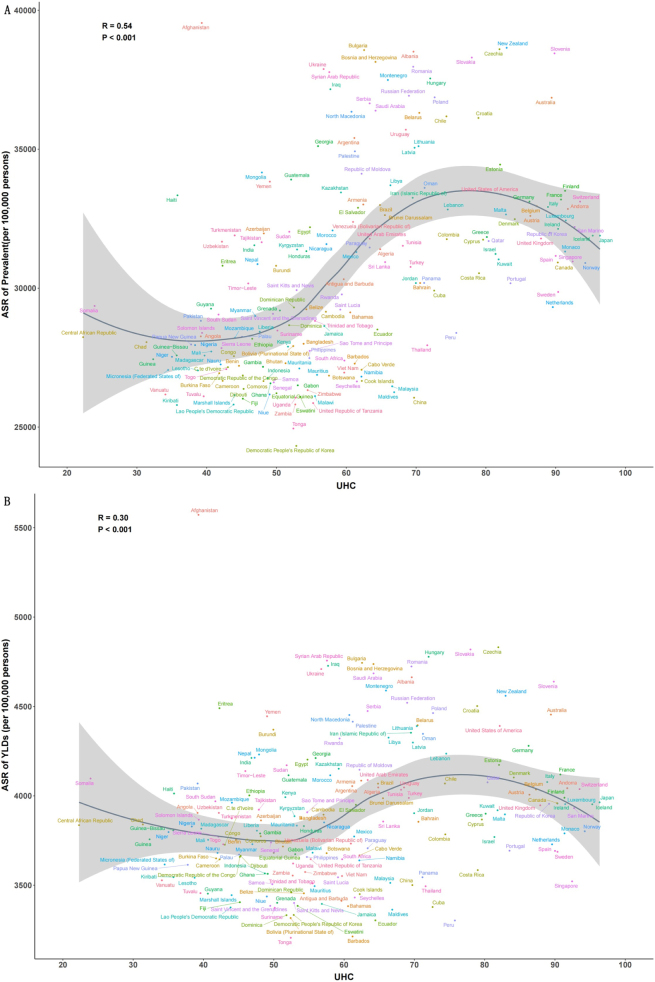
Associations between age-standardized rates and universal health coverage (UHC) effective index. (A) Age-standardized prevalence rates. (B) Age-standardized YLD rates.

We further used smoothing spline models to explore the nonlinear associations between rehabilitation needs and UHC (Fig. 4). Results indicated that both age-standardized prevalence and YLD rates initially increased with a rising UHC effective index, then plateaued or slightly declined, with inflection points around a UHC effective index of 80. Interestingly, countries with higher-than-expected rehabilitation needs included those with low UHC (e.g. Afghanistan, Haiti, Uzbekistan) as well as those with high UHC (e.g. New Zealand, Slovenia, Australia) (Fig. 4).

**Figure 5.**
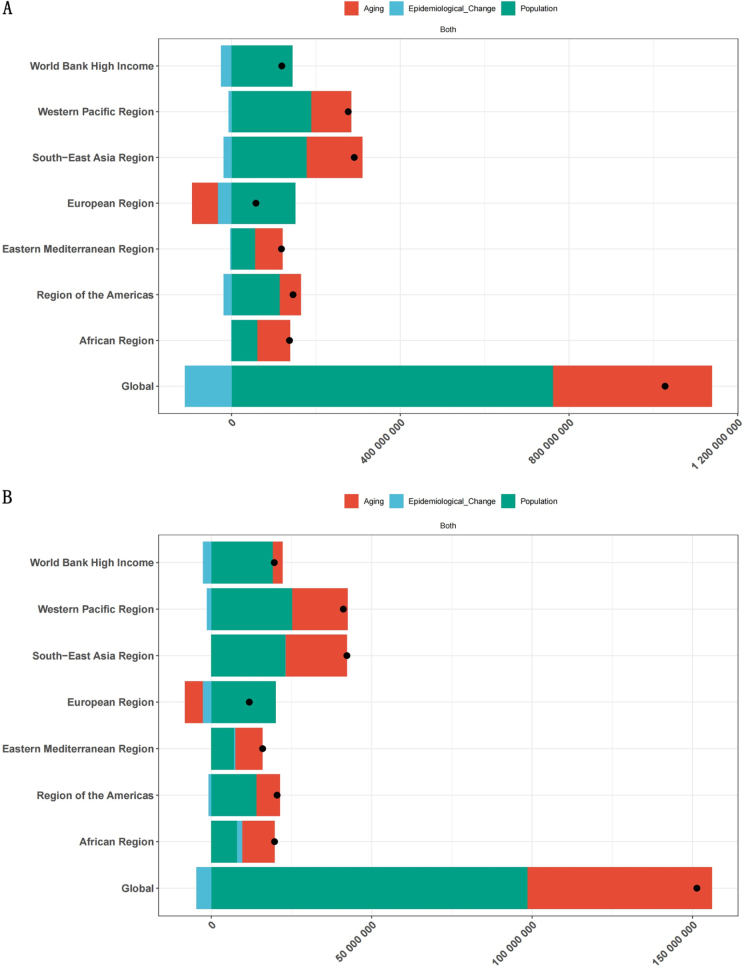
Population-level determinant changes in aging, population growth, and epidemiological changes for prevalence (A) and YLDs (B) of rehabilitation-related conditions globally and in various WHO regions from 1990 to 2021. Black dots represent the total change contributed by all three components.

Between 1990 and 2021, the global increase in rehabilitation needs was primarily driven by population growth and aging. Specifically, the number of people with rehabilitation-related conditions increased by approximately 1.03 billion, with population growth accounting for 74.2% and aging contributing 36.6%. YLDs increased by 151.4 million, with growth and aging contributing 65.1% and 37.98%, respectively (Fig. 5). Regionally, most areas experienced growth driven by both factors. Notably, the African and Eastern Mediterranean regions had the highest relative contribution from aging – 56.22% and 51.16% for prevalence, and 55.74% and 53.61% for YLDs, respectively. In contrast, the rehabilitation needs of other regions were primarily driven by the growth of population. It’s worth noticing that the growth of needs was almost entirely due to population growth in the European regions. Specifically, population growth contributed to the 262.35% increase in prevalence, while the relative contributions of population aging and epidemiological changes were −107.63% and −54.72%, respectively. YLDs were driven by the corresponding contributions, including 169.67% for population growth, −47.85% for aging, and −21.82% for epidemiological changes (Fig. 5). These findings suggested that despite the increase in overall rehabilitation needs, the region has made considerable progress in population structure adjustment and in reducing disease burden.

**Figure 6.**
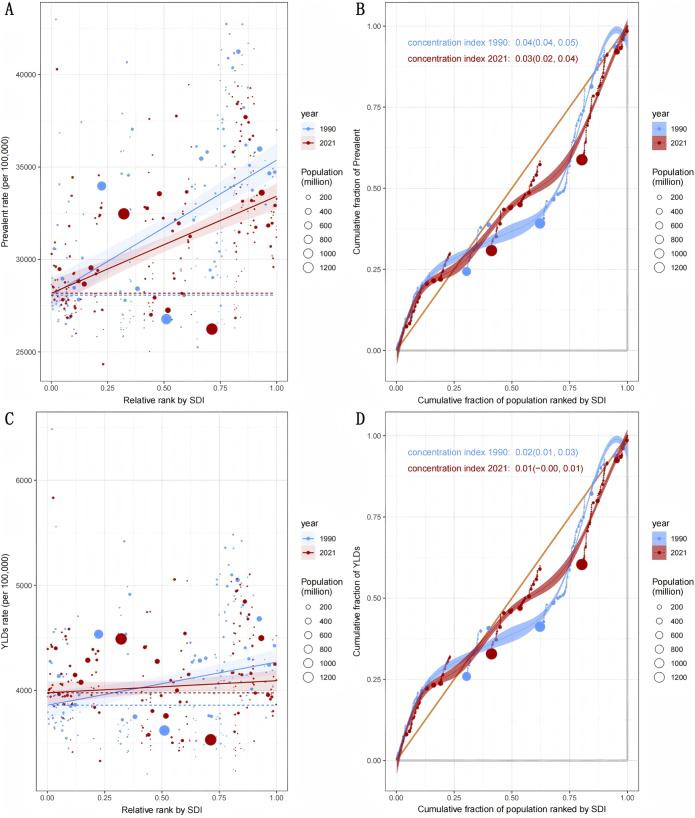
Health inequality regression curves and concentration curves for the prevalence (A and B) and YLDs (C and D) of rehabilitation-related conditions. SDI, Sociodemographic Index.

From 1990 to 2021, although rehabilitation needs remained predominantly concentrated in high-SDI regions, both relative and absolute disparities showed a declining trend (Fig. 6). Specifically, the concentration index for prevalence dropped from 0.04 (95% CI: 0.04–0.05) to 0.03 (95% CI: 0.02–0.04), and the SII from 7309.96 (95% CI: 5494.34–9125.58) to 5248.77 (95% CI: 3909.45–6588.10). For YLDs, the concentration index fell from 0.02 (95% CI: 0.01–0.03) to 0.01 (95% CI: −0.00–0.01), while the SII dropped from 409.96 (95% CI: 191.19–628.73) to 114.88 (95% CI: −75.08–304.84).

**Figure 7.**
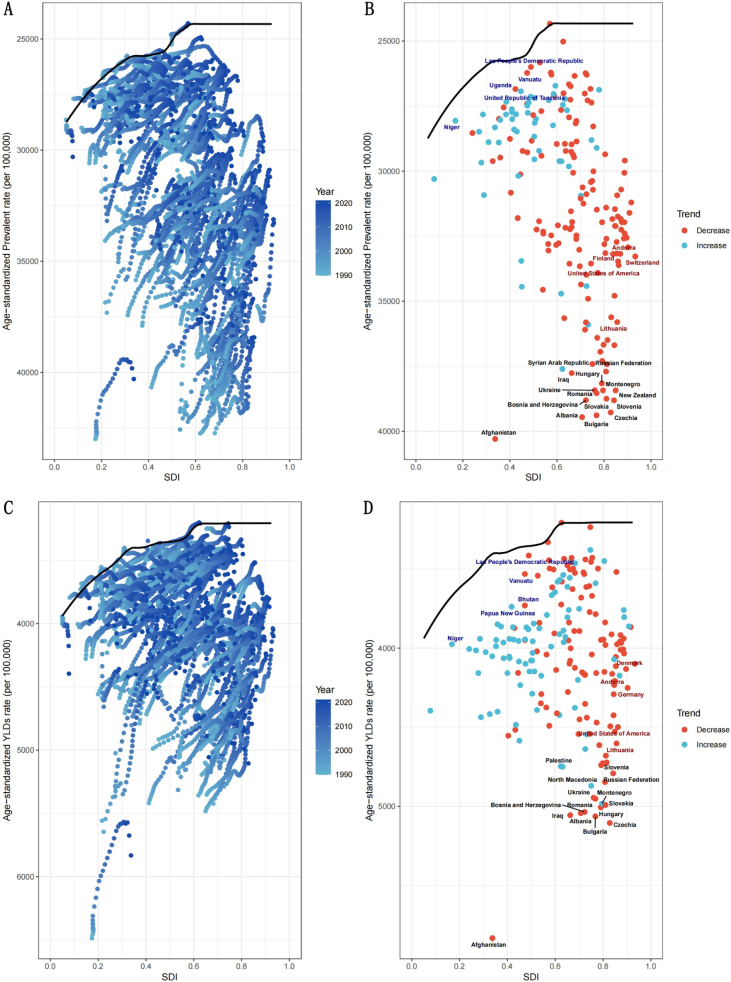
Frontier analysis separately exploring the relationship between SDI and age-standardized prevalence and YLD rates of rehabilitation-related conditions in 195 countries and territories. In (A) and (C), the color change from light blue (1990) to dark blue (2021) represents the change in years. In (B) and (D), each point represents a specific country or territory in 2021, the frontier line is shown in black, and the top 15 countries and territories with the largest differences from the frontier are marked in black. Blue represents low-SDI with the smallest differences from the frontier; red represents high-SDI with the largest differences from the frontier. The direction of age-standardized rate change from 1990 to 2021 is indicated by the color of the dots, with red dots representing decreases and blue dots representing increases. SDI, sociodemographic index.

The frontier analysis showed that as the SDI increased from 0.0 to 1.0, both prevalence and YLDs exhibited a general decline (Fig. 7A and C), suggesting an association between socioeconomic development and the reduction in rehabilitation needs. The 15 countries with the largest gaps from the frontier included the Syrian Arab Republic, Russian Federation, Hungary, Iraq, Montenegro, Ukraine, Romania, New Zealand, Bosnia and Herzegovina, Slovakia, Slovenia, Albania, Bulgaria, Czechia, and Afghanistan. These countries have significant room for improvement in rehabilitation needs. In contrast, low-SDI countries, such as the Lao People’s Democratic Republic, Uganda, Vanuatu, the United Republic of Tanzania, and Niger, showed the smallest gaps, indicating high efficiency of health service under resource-limited conditions. Even among high-SDI countries like Andorra, Finland, Switzerland, the United States, and Lithuania, considerable room for system optimization remains (Fig. 7B and D).

**Figure 8.**
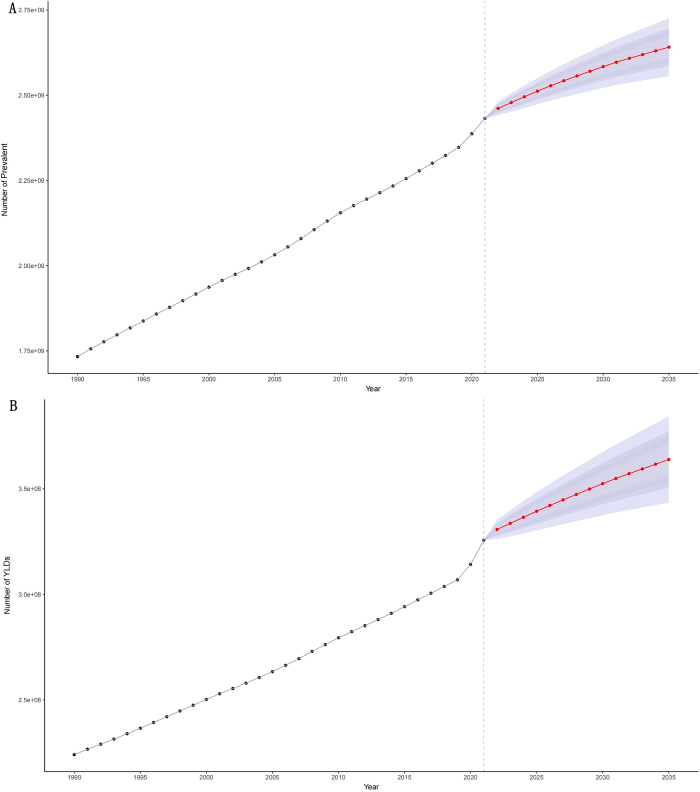
Temporal trends in the number of prevalence (A) and YLDs (B) for rehabilitation-related conditions from 1990 to 2035.

**Figure 9.**
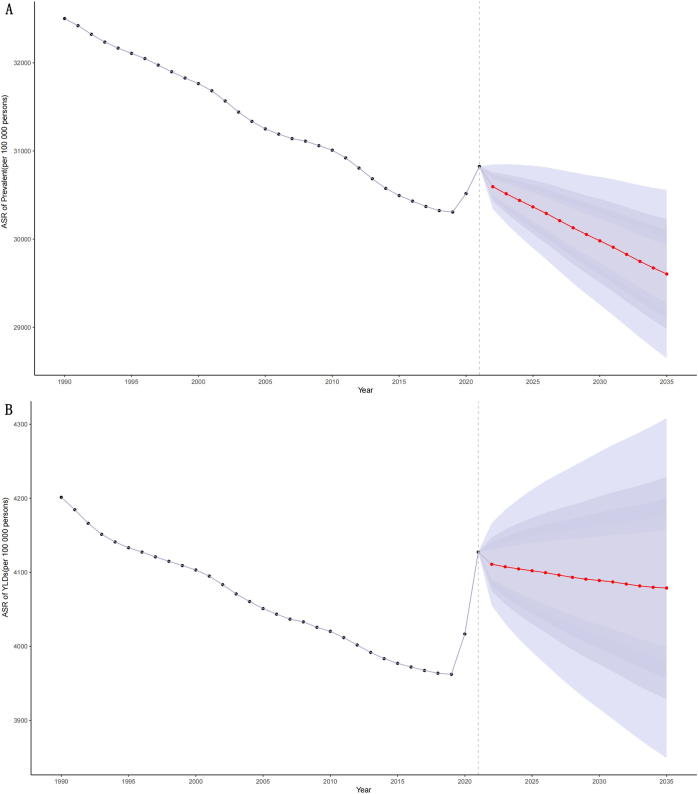
Temporal trends in the age-standardized rates of prevalence (A) and YLDs (B) for rehabilitation-related conditions from 1990 to 2035.

Finally, global rehabilitation needs from 2022 to 2035 were projected using the BAPC model. The results indicated a sustained upward trend in the rehabilitation needs. By 2035, the total population with rehabilitation needs is projected to reach 2.64 (95% UI: 2.56–2.73) billion (Fig. 8A), and YLDs to 364 (95% UI: 344–384) million (Fig. 8B). However, the age-standardized prevalence and YLD rates are expected to show a slow downward trend, with the projected rate in 2035 of 29 604 per 100 000 (95% UI: 28 649–30 559) for prevalence (Fig. 9A) and 4078 per 100 000 (95% UI: 3849–4308) for YLDs (Fig. 9B).

## Discussion

Based on the secondary analysis of the GBD database, this study has systematically assessed the global evolution of rehabilitation needs over the past 30 years by using multi-dimensional methods, providing updated evidence for the guidance of resource allocation. To effectively address the challenge of increasing rehabilitation needs, it is essential to focus on two key strategic directions: first, to reduce the burden of rehabilitation-related conditions in order to ease overall demand; and second, to strengthen rehabilitation service systems to better meet existing needs.

A prerequisite for achieving the first objective is a comprehensive understanding of the current burden of rehabilitation-related conditions and their evolving trends. Our findings have indicated that although the age-standardized prevalence and YLD rates of rehabilitation-related conditions have declined globally from 1990 to 2021, the absolute number has significantly increased due to population growth and aging. By 2021, the global population with rehabilitation-related conditions had reached 2.56 billion, a notable rise from 2.41 billion in 2019, underscoring the growing urgency of rehabilitation needs. The sharp increase in the burden between 2019 and 2021 may be closely related to the outbreak of COVID-19, especially with long COVID now included in GBD 2021 estimates.

Long COVID is defined as symptoms that persist or newly emerge within three months of the initial SARS-CoV-2 infection, last for at least 2 months, and cannot be explained by an alternative diagnosis^[18]^. It can affect various aspects of physical^[19]^, cognitive^[20]^, and psychological health^[21,22]^, leading to rehabilitation needs such as respiratory training, exercise therapy, and cognitive training^[23–25]^. In addition, during the pandemic, disruptions of medical services, poor self-management of chronic diseases, and the accumulation of functional impairments for individuals with conditions further increased the overall demand for rehabilitation services^[26–28]^. These issues highlighted the critical role of rehabilitation in responding to public health emergencies.

In reducing the burden of the pandemic, the application of mRNA vaccines has been proven effective in preventing severe COVID-19 infection, thereby lowering the risk of long-term functional impairment and rehabilitation needs associated with the disease^[29]^. Their protective mechanism relies not only on short-term humoral immunity but also on sustained cellular immune responses, both of which play complementary roles in preventing infection and reducing the risk of severe illness^[30]^. At the same time, sound public health policies and evidence-based vaccine rollout strategies are equally critical. By tailoring vaccination programs to specific populations and regions, the overall disease burden can be reduced, indirectly alleviating the pressure on rehabilitation services^[31]^. However, the ongoing evolution of SARS-CoV-2 may generate new variants that undermine vaccine effectiveness and increase long-term health risks^[32]^. This underscores the need for continuous updates to vaccine strategies, potentially leveraging novel technological platforms (such as nanovaccines) to enhance antigen stability and immune responses^[33]^. In this context, accelerating the integration of rehabilitation into preparedness and response frameworks is essential for addressing the current burden of COVID-19 and enhancing the capacity to manage future global health threats, including zoonotic diseases arising under changing climate conditions^[34]^.

Furthermore, it is crucial to gain a deeper understanding of the sources and regional variations in the burden of rehabilitation-related conditions. Decomposition analysis showed that the increase in rehabilitation needs due to aging was most prominent in the African and Eastern Mediterranean Regions, suggesting that geriatric rehabilitation should be prioritized in the development of rehabilitation services in these areas. Key focus areas may include rehabilitation for osteoarthritis^[35]^, stroke^[36]^, Parkinson’s disease^[37],^ Alzheimer’s disease and dementia^[38]^. In contrast, in the European region, population growth was the sole driver of increased rehabilitation needs. Meanwhile, as shown in Table 1, Europe experienced the largest declines in age-standardized prevalence and YLDs (with EAPCs of −0.40 and −0.37, respectively), further confirming its outstanding performance in elderly care and disease control^[39,40].^ These disparities are evident not only at the regional level but also across different levels of social development. The study found that improvements in social development are associated with a reduction in the burden of rehabilitation-related conditions, although substantial disparities exist among countries with similar SDI levels. For example, countries such as Syria, Russia, Hungary, Iraq, and Ukraine – despite having medium to high SDI levels – have shown poor control over rehabilitation-related disease burdens. This may be attributed to factors such as political instability, slow reconstruction of health systems, shortages of rehabilitation professionals, and inadequate management of chronic conditions, resulting in a large gap between these countries and the “frontier” line. In contrast, some low-SDI countries (e.g. Laos, Uganda, Vanuatu, Tanzania, and Niger) achieved high control efficiency based on their development level, and their experiences may be valuable for other low-income settings. Interestingly, high-SDI countries like Andorra, Finland, Switzerland, the U.S., and Lithuania still show high rehabilitation needs, potentially attributable to population aging, comorbid chronic conditions, high-calorie diets, and sedentary lifestyles. Therefore, addressing the global disparities in rehabilitation burden requires the development of differentiated strategies that consider each country’s population structure, disease profile, level of social development, and national context.

Ultimately, developing practical and effective strategies to control the burden of rehabilitation-related conditions is essential to achieving our goals. Our study demonstrates that when the UHC effective index reaches approximately 80, the burden of rehabilitation-related conditions tends to stabilize or even decline. This finding provides new insights into the relationship between rehabilitation and UHC: rehabilitation services are a vital component of achieving universal health, while improvements in UHC levels contribute to reducing the overall burden of rehabilitation-related conditions. Therefore, accelerating progress toward UHC may be an effective approach to decreasing rehabilitation needs globally. Two key pathways have been identified: first, increasing per capita health expenditure. Evidence suggests that achieving a score of 80 on the UHC effective index, countries would need to reach $1398 pooled health spending per capita (US$ adjusted for purchasing power parity). Second, strengthening service coverage for non-communicable diseases (NCDs). In many countries, service coverage for NCDs remains significantly lagging behind that of communicable diseases and maternal and child health, falling short of meeting the rapidly growing demand for care. Moving forward, enhanced response to NCDs should be prioritized in national health policy agendas^[13]^.

Against the backdrop of rapidly rising global rehabilitation needs, the second objective – building and strengthening rehabilitation service systems – has become an urgent priority. The WHO has initiated several efforts, most notably the “Rehabilitation 2030” initiative^[41–43]^. This framework outlines ten priority areas, including the integration of rehabilitation into UHC, increased financial investment through appropriate mechanisms, and the development of a robust, multidisciplinary rehabilitation workforce tailored to specific national contexts. With the ongoing integration of artificial intelligence and medical technologies, growing evidence suggests that digital and remote rehabilitation may provide viable solutions to many challenges faced by rehabilitation systems, particularly in low-resource settings^[44–48]^. For example, tele-rehabilitation can offer equitable access to high-quality services for patients in remote or underserved areas, while intelligent rehabilitation systems can support primary-level providers in delivering more efficient and accurate care. These innovations have the potential to enhance service coverage, reduce disparities, and contribute to the sustainable development of rehabilitation systems globally.

This study offered the current epidemiological evidence of the rehabilitation field to support the development of evidence-based healthy policies and the optimization of healthy service systems. However, there are still some limitations in this study. First, although this study included 27 diseases, there are more diseases that need rehabilitation intervention in actual clinical practice, such as scoliosis, hemophilia, and lymphedema, which may lead to an overall estimate of rehabilitation needs lower than the actual level. Second, although the data gaps from the lack of original data in some countries were addressed using DisMod-MR estimates^[49]^, the result may not completely reflect the actual condition of some countries, thereby increasing the potential risk of inaccuracy. Finally, rehabilitation needs in many low- and middle-SDI countries might be underestimated due to a lack of high-quality data.

## Conclusions

Overall, although the burden of rehabilitation-related conditions exhibited a gradual decline over the past three decades, some challenges, such as the COVID-19 pandemic, have reversed this trend in recent years. Notably, the burden of rehabilitation-related conditions varies significantly across countries and disease categories, highlighting the need for public health strategies tailored to each country’s specific epidemiological characteristics and population structure. In the future, efforts should focus not only on reducing incidence rates but also on emphasizing long-term rehabilitation management for affected individuals. At the same time, emerging models such as digital rehabilitation and tele-rehabilitation should be further explored to enhance the accessibility and equity of rehabilitation services and to meet the growing global demand.

## Data Availability

The data used in this study were obtained from the WHO Rehabilitation Need Estimator (https://vizhub.healthdata.org/rehabilitation/), which was jointly developed by WHO and IHME based on the Global Burden of Disease Study 2021 (GBD 2021).
